# A study on the efficacy of APACHE-IV for predicting mortality and length of stay in an intensive care unit in Iran

**DOI:** 10.12688/f1000research.12290.1

**Published:** 2017-11-20

**Authors:** Mohammad Ghorbani, Haleh Ghaem, Abbas Rezaianzadeh, Zahra Shayan, Farid Zand, Reza Nikandish

**Affiliations:** 1 Student Research Committee, Shiraz University of Medical Sciences, Shiraz, Iran; 2 Research Center for Health Sciences, Institute of Health, Department of Epidemiology, School of Health, Shiraz University of Medical Sciences, Shiraz, Iran; 3 Department of Epidemiology, School of Health, Shiraz University of Medical Sciences, Shiraz, Iran; 4 Trauma Research Center, Department of Community Medicine, School of Medicine, Shiraz University of Medical Sciences, Shiraz, Iran; 5 Anesthesiology and Critical Care Research Center, Shiraz University of Medical Sciences, Shiraz, Iran; 6 Department of Emergency Medicine, Shiraz University of Medical Sciences, Shiraz, Iran

**Keywords:** APACHE-IV, Mortality, Length of stay, Intensive care unit, emergency

## Abstract

**Background: **Clinical assessment of disease severity is an important part of medical practice for prediction of mortality and morbidity in Intensive Care Unit (ICU). A disease severity scoring system can be used as guidance for clinicians for objective assessment of disease outcomes and estimation of the chance of recovery. This study aimed to evaluate the hypothesis that the mortality and length of stay in emergency ICUs predicted by APACHE-IV is different to the real rates of mortality and length of stay observed in our emergency ICU in Iran.

**Methods: **This was a retrospective cohort study conducted on the data of 839 consecutive patients admitted to the emergency ICU of Nemazi Hospital, Shiraz, Iran, during 2012-2015. The relevant variables were used to calculate APACHE-IV.  Length of stay and death or discharge, Glasgow coma score, and acute physiology score were also evaluated. Moreover, the accuracy of APACHE-IV for mortality was assessed using area under the Receiver Operator Characteristic (ROC) curve.

**Results: **Of the studied patients, 157 died and 682 were discharged (non-survivors and survivors, respectively). The length of stay in the ICU was 10.98±14.60, 10.22 ± 14.21 and 14.30±15.80 days for all patients, survivors, and non-survivors, respectively. The results showed that APACHE-IV model underestimated length of stay in our emergency ICU (p<0.001). In addition, the overall observed mortality was 17.8%, while the predicted mortality by APACHE-IV model was 21%. Therefore, there was an overestimation of predicted mortality by APACHE-IV model, with an absolute difference of 3.2% (p=0.036).

**Conclusion: **The findings showed that APACHE-IV was a poor predictor of length of stay and mortality rate in emergency ICU. Therefore, specific models based on big sample sizes of Iranian patients are required to improve accuracy of predictions.

## Introduction

Clinical assessment of disease severity is an important part of medical practice to predict mortality and morbidity in Intensive Care Unit (ICU) ^1^. An acceptable goal in ICU is saving the lives of critically ill patients, since not all patients admitted to an ICU have a normal life after leaving and some will not survive because of disease severity ^2^.

Specialties of ICUs should predict patient outcomes to focus more on efficient use of ICU beds for critically ill patients ^2^. Disease severity scoring systems can be used as a guidance for clinicians in the objective assessment of disease outcomes and estimation of the chance of recovery ^2^. Acute Physiology And Chronic Health Evaluation (APACHE), introduced in 1981, considers various parameters, including vital signs, physiological variables, neurological score, urine output, age, and comorbid conditions ^3^. The latest version of APACHE-IV is calculated based on 129 variables derived within the first 24 h of ICU admission ^1^, which were assessed from over 110,588 patients admitted to more than 104 ICUs across the USA ^4, 5^. Some studies have suggested the superior advantage of APACHE-IV compared to other risk scoring systems ^6, 7^.

Evaluation of clinical outcomes and effectiveness of care in ICU patients is influenced by predictive scoring models that compute measures of disease severity and the associated probability of death. APACHE is a logistic regression model involving both physiological and laboratory parameters. It is a commonly used ICU stratification instrument, which is known as an accurate predictor of mortality. Yet, model accuracy decreases over time and requires updating occasionally. A study conducted in 2012 indicated that APACHE-III performance was inadequate even with a predicted mortality of only 2% higher than the observed mortality rate (16% vs. 14%) ^8^. A similar study conducted on APACHE-IV showed that the ICU’s outcome prediction by the model is different to observed values in clinical setting between the predicted and the observed mortality rate ^9^.

To our knowledge, no study has been conducted to evaluate the accuracy of APACHE-IV for predicting mortality and length of stay in emergency ICUs in Iran. This study aimed to evaluate the hypothesis that the mortality and length of stay in emergency ICUs predicted by APACHE-IV is different than that observed in reality.

## Methods

This was a retrospective cohort study conducted on the medical records of 839 consecutive patients admitted in the emergency ICUs in Nemazi Hospital, Shiraz, Iran, between July 2012 and July 2015. The patients of this study were selected from all patients referred to the ICUs of the Center during the study period using convenient sampling method. The total number of patients admitted during this period was 839. The inclusion criterion was minimum 24 hour admission in the ICU and there was no exclusion criterion for this study. The Namazi Hospital is a tertiary referral hospital affiliated to Shiraz University of Medical Sciences, Shiraz, Iran. All the experimental procedures and study protocol of the study were approved by the local ethics committee of Shiraz University of Medical Sciences (protocol no. 94-7636), which were in complete accordance with the ethical standards and regulations of human studies of the Declaration of Helsinki (2014).

The medical records of 839 consecutive patients admitted to the emergency ICUs of Nemazi Hospital were analyzed. The variables used to calculate APACHE-IV score included age, sex, dates of admission, discharge or death, systolic and diastolic blood pressure, body temperature, heart rate, respiratory rate, glucose, blood urea nitrogen, serum sodium, creatinine, blood hematocrit, white blood cells, serum albumin and bilirubin, urine output during the first 24 h of ICU admission, pH, fraction of inspired oxygen, partial pressure of carbon dioxide, partial pressure of oxygen, and bicarbonate ^5^.

Death or discharge and length of stay in ICU were followed up by referring to patients’ medical records. Additionally, APACHE-IV score, Glasgow coma score (GCS), and acute physiology score (APS) were calculated according to www.cerner.com (the authors registered as a user in order to calculate all the parameters).

### Statistical analysis

Qualitative variables were expressed as number and percentage, and quantitative variables as mean ± standard deviation. Student’s t-test, Mann–Whitney U, Wilcoxon rank test, and Chi-square tests were used where appropriate to compare survivors and non-survivors regarding demographic and clinical variables. In addition, Spearman’s correlation coefficient was used to examine the relationship between APACHE-IV score and length of stay in ICU. Finally, accuracy of APACHE-IV for mortality was assessed using area under the Receiver Operator Characteristic (ROC) curve with an attribution of ‘good’ > 0.80. The data are expressed as mean ± SD for all variables. All statistical analyses were carried out using Stata (version 13, Windows). As the distribution of the quantitative variables was not normal, Mann Whitney U test was used for comparisons of the difference between the survivor and non-survivor groups. For the sex variable the Chi-square test was used. p ≤ 0.05 was considered to be statistically significant.

## Results

This study was conducted on 839 patients among whom, 157 died and 682 were discharged (non-survivors and survivors, respectively). The length of stay in ICU was 10.98±14.60, 10.22±14.21, and 14.30±15.80 days in all patients, survivors, and non-survivors, respectively. Demographic information and the clinical features of the patients are summarized in Table 1.

**Table 1.** Demographic and clinical features of the 839 patients. For the comparisons of quantitative variables Mann Whitney U test was used and for the sex variable the Chi square test was used. P < 0.05 represents significant difference between the survivor and non-survivor groups.

Characteristics	Total (n=839)	Survivors (n=682)	Non-survivors (n=157)	P-value
Age (years), mean ± SD	48.83±19.65	46.72±19.05	58.03±19.61	0.001
Sex, n (%) *Male* *Female*	452 (53.9) 387 (46.1)	374 (82.7) 308 (79.6)	78 (17.3) 79 (20.4)	0.243
ICU length of stay (days), mean ± SD	10.98±14.60	10.22±14.21	14.30±15.80	0.001
Glasgow coma score, mean ± SD	10.43±4.14	11.03±3.93	7.77±4.00	0.001
APACHE-IV score, mean ± SD	52.93±29.48	46.34±22.88	81.81±36.99	0.001
Acute physiology score, mean ± SD	51.65±29.21	45.06±22.50	80.52±36.86	0.001

The results showed no significant difference between the two groups regarding sex (p=0.243). However, the two groups were significantly different with respect to the means of age (p≤0.001), ICU length of stay (p≤0.001), GCS (p≤0.001), APACHE-IV score (p≤0.001), and APS (p≤0.001) ( Table 1).

### Evaluation of APACHE-IV score

Outcome variables have been summarized in Table 2. Accordingly, mean ± SD of observed length of stay in ICU was 10.98±14.60 days. However, predicted ICU length of stay by the APACHE-IV model was 5.43±2.50 days (p<0.001). This indicated that APACHE-IV underestimated ICU length of stay in our emergency ICU. Additionally, the overall observed mortality was 17.8%, while the predicted mortality by APACHE-IV was 21%. Thus, mortality was overestimated by APACHE-IV model with an absolute difference of 3.2% (p=0.036).

**Table 2.** Outcome variables for the total cohort (n=839). For the comparisons of stay length Mann Whitney U test was used and for the mortality rate the Chi square test was used. P < 0.05 represents significant difference between the survivor and non-survivor groups.

Characteristics	Observed	Predicted	P-value
ICU length of stay, mean ± SD	10.98±14.60	5.43±2.50	<0.001
Mortality, n (%)	157 (17.8)	177 (21.0)	0.036

**Figure 1.**
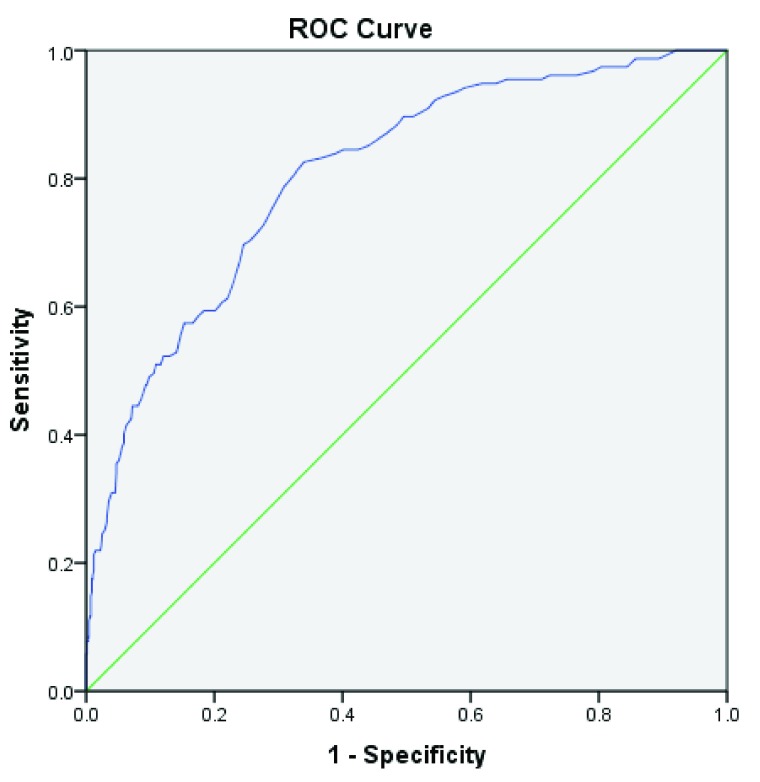
ROC curve for APACHE-IV score and observed mortality.

ROC curve for APACHE-IV score and observed mortality has been depicted in Figure 1. Accordingly, area under the curve of the APACHE-IV score was 0.81, 95% CI (0.77, 0.84). These values were statistically significant and could be an appropriate predictor for observed mortality. Nevertheless, there was a significant weak correlation between APACHE-IV score and observed ICU length of stay (r=0.175, p<0.0001).

Data for the study on efficacy of APACHE-IV for predicting mortality and length of stay in an intensive care unit in Iran Click here for additional data file. Copyright: © 2017 Ghorbani M et al. 2017 Data associated with the article are available under the terms of the Creative Commons Zero "No rights reserved" data waiver (CC0 1.0 Public domain dedication).

## Discussion

A retrospective cohort study was conducted among 839 patients referred to ICU at Namazi Hospital in Shiraz, Iran. The study results showed that APACHE-IV underestimated the length of stay in our emergency ICU. In addition, the overall observed mortality was 17.8%, while the predicted mortality by APACHE-IV was 21%. Thus, there was an overestimation of predicted mortality by APACHE-IV, with an absolute difference of 3.2% (p=0.036).

Several factors may contribute to poor performance of APACHE-IV in emergency ICU. APACHE-IV is a good benchmark to determine disease severity; however, the present study results indicated that it did not function well to predict the risk of mortality and length of stay in emergency ICU. Other studies also reported this score not to be predictive of mortality ^10, 11^. The poor estimate may be attributed to various reasons. Firstly, the estimations were achieved based on American rather than our own patients’ data. Generally, predictive scoring systems function appropriately in populations where scores are derived from the same population data. Therefore, many experts recommend external validation at national, regional, or institutional levels. For example, APS3 has several customized versions for seven geographic regions ^12, 13^.

Secondly, in America, where APACHE was calibrated, patients go from ICU to ‘step down’, a halfway ward, before moving to general wards. In Iran, patients directly go to general wards, and consequently, they have to stay in ICUs for a longer time period than American patients.

Thirdly, even if scores are achieved by patients’ data, they must be calibrated over time. This is because case-mix varies, quality of care improves, and types of disease changes over time. In general, accurate calibration is a key characteristic that should be ensured for all risk scoring systems. Calibration may weaken over time, especially due to the effects of altered patient interventions and case-mix. This often results in overestimation of death or mortality ^14^.

The findings of the present study revealed that APACHE-IV score based on our data would be an appropriate predictor for the observed mortality, while this relationship was not confirmed by the APACHE-IV score according to the American database ^9^. Moreover, our findings showed a similar relationship between APACHE-IV score and ICU length of stay with the study conducted on the United States database ^9^. Overall, a large patient’s database should exist in order for APACHE-IV to correctly predict outcomes (i.e. mortality and ICU length of stay).

### Strengths and limitations of the study

The strength of this study should be noted. This study is the first study in Iran that demonstrated that predictions of mortality and ICU length of stay should be based on data obtained from Iranian and not American patients. However, this study had some limitations, the first of which being the intrinsic shortcomings of its retrospective design (inability to confirm causation, and dependence on medical records). Another study limitation was its small sample size; however, to date, our cohort of 839 patients is the largest reported study of patients admitted to emergency ICUs in Iran.

In conclusion, the findings of this study suggested that the American based APACHE-IV score is a poor predictor of length of stay and mortality in emergency ICU in Iran. Therefore, specific models based on big sample sizes of our patients from Iran are required to improve the accuracy of predictions of mortality and ICU length of stay for our country.

## Data availability

The data referenced by this article are under copyright with the following copyright statement: Copyright: © 2017 Ghorbani M et al.

Data associated with the article are available under the terms of the Creative Commons Zero "No rights reserved" data waiver (CC0 1.0 Public domain dedication). 

Dataset 1: Data for the study on efficacy of APACHE-IV for predicting mortality and length of stay in an intensive care unit in Iran. doi, 10.5256/f1000research.12290.d177987 ^15^
