# Supplementary material for: A study on the efficacy of APACHE-IV for predicting mortality and length of stay in an intensive care unit in Iran
Source: F1000Res. 2017 Nov 20;6:2032. [Version 1] doi: 10.12688/f1000research.12290.1 (PMC5710303; doi:10.12688/f1000research.12290.1)
Supplement: Data for the study on efficacy of APACHE-IV for predicting mortality and length of stay in an intensive care unit in Iran [file f1000research-6-13304-s0000.tgz › 698bcabe-e75d-42f2-bc56-dbdd94b2bc25_Dataset_1.zip!/Dataset 1/Descriptions of the variables of the dataset.docx]

Table2-dataset- The descriptions of the variables of the dataset

| **Variables** | Description | Comments |
| --- | --- | --- |
| Age |  |  |
| Sex | 1=male  2=female |  |
| Temperature L | Temperature ( ⁰C) L |  |
| Temperature H | Temperature ( ⁰C) H |  |
| SystolicBPL | Systolic blood pressure (mm/Hg) L |  |
| SystolicBPH | Systolic blood pressure (mm/Hg) H |  |
| DiastolL | Diastolic blood pressure (mm/Hg) L |  |
| DiastolH | Diastolic blood pressure (mm/Hg) H |  |
| HeartRateL | Heart rate ( /min) L |  |
| HeartRateH | Heart rate ( /min) H |  |
| RespiratoryRateL | Respiratory rate ( /min) L |  |
| RespiratoryRateH | Respiratory rate ( /min) H |  |
| SodiumL | Sodium (m mol/L) L |  |
| SodiumH | Sodium (m mol/L) H |  |
| GlucoseL | Glucose L |  |
| GlucoseH | Glucose H |  |
| CreatinineL | Creatinine L |  |
| CreatinineH | Creatinine H |  |
| BUNL | Blood urea nitrogen (BUN ) L |  |
| BUNH | Blood urea nitrogen (BUN ) H |  |
| HCTL | Hematocrit (HCT) % L |  |
| HCTH | Hematocrit (HCT) % H |  |
| WBCL | White blood cells (WBC), (x103/mm3) L |  |
| WBCH | White blood cells (WBC), (x103/mm3) H |  |
| Fio2 | Fraction of inspired oxygen (Fio 2) % |  |
| PH | potential hydrogen (PH) |  |
| Po2 | Partial pressure of oxygen (pO2) |  |
| PCO2 | Partial pressure of carbon dioxide (pCO2) |  |
| Hco3 | Bicarbonate (Hco3) |  |
| Urineoutput | Urine output during the first 24 hour of ICU stay (ml/24hrs) |  |
| Albumin | Serum Albumin |  |
| Bilirubin | Bilirubin |  |
| K | Potassium (K) |  |
| Spontaneous | * | For Glasgow Coma Score calculation |
| Tospeech | * | For Glasgow Coma Score calculation |
| Topain | * | For Glasgow Coma Score calculation |
| Absent | * | For Glasgow Coma Score calculation |
| Conversesamporiented | * | For Glasgow Coma Score calculation |
| Conversesampdisoriented | * | For Glasgow Coma Score calculation |
| Inappropriate | * | For Glasgow Coma Score calculation |
| Incomprenensible | * | For Glasgow Coma Score calculation |
| Absent-A | * | For Glasgow Coma Score calculation |
| Obey | * | For Glasgow Coma Score calculation |
| Localizes | * | For Glasgow Coma Score calculation |
| Withdraws | * | For Glasgow Coma Score calculation |
| Decorticateflexion | * | For Glasgow Coma Score calculation |
| Decorticateextension | * | For Glasgow Coma Score calculation |
| Absent-B | * | For Glasgow Coma Score calculation |
| Glasgow Coma Score | * |  |
| CRF | yes=1 | Presence of chronic disease |
| Aids | No=0 | Presence of chronic disease |
| Hepatic | yes=1 | Presence of chronic disease |
| Lymphoma | No=0 | Presence of chronic disease |
| Metastatic | yes=1 | Presence of chronic disease |
| Leukemia | No=0 | Presence of chronic disease |
| Immunosuppression | yes=1 | Presence of chronic disease |
| Cirrhosis | No=0 | Presence of chronic disease |
| Previous | Previous ICU LOS (days) |  |
| Postoperative | ICU admission after surgery  yes=1  No=0 |  |
| Emergency | Emergency Surgery  yes=1  No=0 |  |
| Readmission | yes=1  No=0 |  |
| ventilated | Ventilated at any time (first 24 hrs) |  |
| APACHEIVScore | Acute Physiology and Chronic Health Evaluation Score |  |
| APSScore | Acute Physiology Score | Acute Physiology Score, score calculated without taking into account patient's age and Chronic Health Condition details. |
| Icu.los | Length of stay in ICU |  |
| Live | Patient released alive=1  Patient died=2 |  |

Notes: L: lowest, H: highest, yes=1, No=0,
