# Supplementary material for: A study on the efficacy of APACHE-IV for predicting mortality and length of stay in an intensive care unit in Iran
Source: F1000Res. 2017 Nov 20;6:2032. [Version 1] doi: 10.12688/f1000research.12290.1 (PMC5710303; doi:10.12688/f1000research.12290.1)
Supplement: Peer review history

# Peer review: Referee response for version 1

**DOI:** 10.5256/f1000research.13304.r28373

**Published:** 2017-11-27

**Authors:** Ali Yadollahpour

**Affiliations:** 1 Department of Medical Physics, Faculty of Medicine, Ahvaz Jundishapur University of Medical Sciences, Ahvaz, Iran; 2 Bioelectromagnetic Clinic, Ahvaz Imam Khomeini Hospital, Ahvaz, Iran

The manuscript entitled " A study on the efficacy of APACHE-IV for predicting mortality and length of stay in an intensive care unit in Iran" was reviewed. This is a well conducted study with appropriate methodology. The data provided are of good volume and with enough details. The sample size is of particularly important resulting in the more robust evidence. The authors could appropriately discussed the results and compared their findings with the similar conducted studies. Conducting further studies in the country with big sample size in the ICU patients can shed further lights to adopt appropriate policies for improving the healthcare conditions and reducing the mortality rate of ICU patients.

I have read this submission. I believe that I have an appropriate level of expertise to confirm that it is of an acceptable scientific standard.

# Peer review: Referee response for version 1

**DOI:** 10.5256/f1000research.13304.r28177

**Published:** 2017-11-22

**Authors:** Nelson Elias, Jose Eduardo Grandi Ribeiro

**Affiliations:** 1 Vila Velha Hospital, Vila Velha, Brazil

Classification:

 Research - retrospective cohort study with a critical review of the corresponding literature in Apache IV scoring systems

 Originality and Relevance:

 a)         Pertinent for the journal - F1000Research

 b)         Original

 c)         Contribution towards the development of specific models based in the characteristics of Iranian population

 Content

 a          Coherence between the paper’s title and its content

 b          Coherence between the paper’s summary and its content

 c          The article fulfils its stated objectives

 d          The conclusion is adequate

 e          Relevant and current bibliographic references (I didn’t find in the text the reference 15)

 The discussion of the topic is ordered, didactic, clear and concise

 The article should be published in the journal with slight modification (include reference 15 in the text)

We have read this submission. We believe that we have an appropriate level of expertise to confirm that it is of an acceptable scientific standard.
